# The Effect of Midwife-Led Continuous Labor Support Care on Delivery Mode and Timely Initiation of Breastfeeding in Primigravida Women in Northwest Ethiopia: A Hybrid Type I Implementation Study

**DOI:** 10.3390/ijerph23040428

**Published:** 2026-03-29

**Authors:** Mengstu Melkamu Asaye, Getie Mihret Aragaw, Eden Bishaw Taye, Kihinetu Gelaye Wudineh, Sara Bayes

**Affiliations:** 1 Department of Women and Family Health, School of Midwifery, College of Medicine and Health Sciences, University of Gondar, Gondar P.O. Box 196, Ethiopia; 2 Department of General Midwifery, School of Midwifery, College of Medicine and Health Sciences, University of Gondar, Gondar P.O. Box 196, Ethiopia; getie.mhret@uog.edu.et; 3 Department of Clinical Midwifery, School of Midwifery, College of Medicine and Health Sciences, University of Gondar, Gondar P.O. Box 196, Ethiopia; eden.bishaw@uog.edu.et; 4 Department of Midwifery, College of Medicine and Health Sciences, Bahir Dar University, Bahir Dar P.O. Box 79, Ethiopia; kihnetu.gelaye@bdu.edu.et; 5 School of Nursing and Midwifery, Edith Cowan University, Joondalup 6027, Australia; s.bayes@ecu.edu.au; 6 School of Nursing, Midwifery and Paramedicine, Australian Catholic University, Melbourne 3065, Australia; 7 Fiona Stanley Hospital, 14 Barry Marshall Parade, Murdoch 6150, Australia

**Keywords:** midwife-led, continues labor support, childbirth, breastfeeding, Ethiopia

## Abstract

**Highlights:**

**Public health relevance—How does this work relate to a public health issue?**

- Midwife-led continuous labor support increases spontaneous vaginal delivery rates and reduces episiotomies.
- Midwife-led continuous labor support promotes early breastfeeding, benefiting newborn health.

**Public health significance—Why is this work of significance to public health?**

- Midwife-led continuous labor support reduces unnecessary obstetric interventions.
- Enhancing early initiation of breastfeeding supports neurodevelopment in neonates.

**Public health implications—What are the key implications or messages for practitioners, policy makers and/or researchers in public health?**

- Scaling up midwife-led continuous labor support increases spontaneous vaginal delivery rates, reduces episiotomy rates, and promotes early breastfeeding.
- Midwife-led continuous labor and childbirth support improves childbirth outcomes.

**Abstract:**

Midwife-led continuous labor and childbirth support reduces the episiotomy rate and increases the rates of vaginal delivery and early initiation of breastfeeding. However, no studies have yet been conducted in Ethiopia. This study aimed to evaluate the effect of midwife-led continuous labor and childbirth support. A quasi-experimental study involving 419 primigravida women compared an intervention given by trained midwives at two hospitals to routine at two others. Data were collected from July to December 2024 through interviews and record reviews. The McNemar test was used to compare changes, with a *p*-value of less than 0.05. Midwife-led continuous labor support increased the rate of spontaneous vaginal delivery among primigravida women from 55.8% in the control group to 81.2% in the intervention group, resulting in a net increase of 25.4%. The intervention reduced the episiotomy rate from 25.2% in the control group to 16.4% in the intervention group, with a net reduction of 8.8%. It also increased early initiation of breastfeeding from 56.8% in the control group to 73.3% in the intervention group, with a net increase of 16.5%. Midwife-led continuous labor and childbirth support was effective in this study. The intervention enhances positive childbirth outcomes and could be implemented and sustained.

## 1. Introduction

Childbirth is a crucial moment in a woman’s life, with far-reaching consequences for her physical and psychological well-being, as well as that of her newborn [1]. Physiological and psychological support promotes natural hormone production and the natural childbirth process. This can help avoid unnecessary interventions such as vacuum extraction, cesarean section, oxytocin administration, and disrespectful care during the birthing process [2]. To support empowering birth, the World Health Organization (WHO) recommends that all women have access to midwife-led continuous labor and childbirth support. If every woman has access to a trusted person who provides emotional, psychological, and practical support at all times, the birth experience and outcomes can be improved [3].

Childbirth ceremonies and the sharing of experiences are of great importance in Ethiopian culture. They encourage women to have a positive birth experience, even though these practices have not yet been implemented in health facilities. The implementation of midwife-led continuous labor and childbirth support requires more attention and action.

The availability of a labor waiting room in facilities, unemployed midwives in the county, community support for women in labor, and accompanying women to health facilities all represent opportunities for implementing midwife-led continuous labor support in Ethiopia. These strategies can facilitate vaginal birth compared to vacuum or forceps delivery and cesarean section and reduce episiotomy. They could also encourage primigravida women to initiate breastfeeding early [4].

Midwife-led continuous labor and childbirth support promotes labor progress, increases the rate of vaginal delivery, and reduces repeated unnecessary obstetric interventions [5]. It also reduces complications such as obstetric bleeding, infection, perineal laceration, and negative childbirth experiences related to instrumental and cesarean section deliveries [6]. However, midwife-led continuous labor support has become the exception rather than the rule in hospitals worldwide for pregnant women during labor. Concerns about the dehumanization of women’s birth experiences have led to calls for a return to preferential continuous midwife-led support for women during labor [7].

The implementation of continuous labor and childbirth support by midwives, including unemployed midwives in health facilities, offers a valuable opportunity to enhance continuously supported care [7]. This approach aligns with global calls to improve positive birth experiences and outcomes. Challenges such as unnecessary obstetric interventions in health facilities and home births without skilled attendance require urgent attention. These issues could be addressed by integrating midwife-led continuous support strategies that are framework-based, contextualized, and culturally appropriate. Evidence suggests that this is a cost-effective and beneficial practice, particularly in Ethiopia. Therefore, the study aimed to evaluate the effect of midwife-led continuous labor and childbirth support on mode of delivery and early initiation of breastfeeding in Ethiopia in 2024.

## 2. Methods and Materials

### 2.1. Study Design

The study employed a comparative quasi-experimental design at four hospitals in Ethiopia: two intervention hospitals and two control hospitals. The selected hospitals are located in different zones within the region, with buffer zones considered to prevent information contamination. The distance between the intervention and control hospitals is more than 90 kilometers. Study participants were proportionally allocated to the continuous labor support intervention and control care hospitals.

### 2.2. Setting

The research was conducted in four public hospitals in the Amhara region of northwest Ethiopia: Debark General Hospital, Debat Primary Hospital, Addis Zemen Primary Hospital, and Dembia Primary Hospital. Each hospital has a maternity ward comprising triage, first stage, second stage, and postnatal units. Midwives, physicians, and emergency surgeons work in these wards. The average monthly birth rate at each hospital ranged from 120 to 240.

### 2.3. Sample Size

The primary outcome of the study was vaginal mode of delivery, while episiotomy and early initiation of breastfeeding were secondary outcomes. The sample size was calculated based on the proportion of vaginal deliveries in the intervention group (P1 = 0.75) and the control group (P2 = 0.52) [8], with a desired effect size of 0.48, alpha = 0.05, power = 0.80, and an attrition rate of 20%. Midwife-led continuous labor support increases spontaneous vaginal birth and reduces instrumental or cesarean section deliveries. This was the aim of the project, and we used this reference as it is more closely related to our objective. The effect size was calculated and taken from the previous study. It is better to consider this when comparing future studies with it. The total sample size was 432 (216 in the intervention group and 216 in the control hospitals).

### 2.4. Participants

The study included primigravida women at 37–42 weeks of gestation, with singleton pregnancies, cephalic presentation, regular uterine contractions, and in the first stage of labor. The exclusion criteria were pregnant women with cardiovascular disease, diabetes, kidney disease, mental disorders, preeclampsia, placenta previa, fetal death, or fetal distress.

### 2.5. Variables

The outcome variables measured in this study were mode of delivery (spontaneous vaginal delivery, instrumental delivery [vacuum/forceps], or cesarean section), episiotomy (yes/no), and initiation of breastfeeding within one hour (early) or after more than one hour (late). The independent variables, each with various categories, were maternal age, religion, marital status, residence (urban/rural), education, occupation, and family monthly income.

### 2.6. Recruitment

Recruitment took place at the labor triage site in the maternity wards. Only primigravida women who met the eligibility criteria and arrived in the delivery room of the study hospitals in the first stage of labor were further briefed and assessed for eligibility. Trained senior midwives working in the selected hospitals conducted this process. Eligible primigravida women received the participants’ information sheet from trained midwives. Those who volunteered and were interested in participating in the study signed a written consent form.

### 2.7. Interventions

The midwife-led continuous labor support intervention includes, physical support (such as comforting touch, massage, dynamic birth positions, and assistance), emotional support (continuous presence, reassurance, and praise), explanation of procedures, and assistance in making informed decisions [9,10]. It also involves labor pain coping techniques and advocacy (helping the woman articulate her wishes to others, as well as pain relief coping strategies) [11]. The intervention includes breathing exercises with intervals of more than 20 min, 20–40 min of continuous back massage over the lower limbs and lower back, physical movement, and allowing various birthing positions. The duration of the breathing exercises, massage, and trying different birth positions varies. The intervention was provided frequently, with the total time required being more than 1–2 h for women in labor.

The midwives allowed the primigravida women to change position during the intervention. Midwife-led continuous labor support (*n* = 213) was provided to primigravida women in the intervention group by trained midwives, starting from the first to the second stages of labor. Primigravida women in the routine care group (*n* = 206) received standard care, including the lithotomy birthing position and only minimal physical support, rather than massage and reflection to reduce labor pain. Minimal reassurance and praise were given to women during labor and birth by midwives who had not received any of the study training in the two control hospitals.

Midwife-led continuous labor support care training was provided to all midwives working in the two intervention hospitals, as well as to two unemployed midwives at the intervention sites, who received two days of training. The training included theoretical sessions, role plays, video presentations, and a half-day of clinical practice demonstrations.

Additional midwives were hired and trained for two days on the questionnaire and the collection of outcome variables. The training was delivered by the principal investigator and one of the co-investigators. It also emphasized the importance of maintaining the privacy and confidentiality of study participants. Each data set was assigned an anonymous, computer-generated four-digit number. These midwives also assisted other midwives.

### 2.8. Data Collection Techniques

Data were collected between July and December 2023 through face-to-face interviews and medical record reviews, in accordance with the Declaration of Helsinki (1975, revised 2013) [12]. In line with this declaration, ethical approval was obtained from the Institutional Review Board (IRB) of the University of Gondar (Ref. VP/RTT/05/931/2023) prior to the study, ensuring adherence to both national and international guidelines. Primigravida women who volunteered and provided written informed consent were interviewed about their socio-demographic characteristics at the beginning of admission, and outcome-related variables were assessed in their medical records before discharge from the maternity wards. Data were collected by trained midwives.

### 2.9. Statistical Analysis

The collected data were manually checked for completeness and consistency. EpiData version 7 software was used to enter, clean, and code the data. The data were then exported to SPSS version 20 software for analysis. Continuous and categorical numerical data were presented as frequencies and percentages. All results are shown in tables. The effect of the intervention was assessed using the McNemar test between the intervention and control groups, with a *p*-value of less than 0.05 considered statistically significant. Multivariate logistic regression models for mode of delivery and early initiation of breastfeeding were used to determine the contributions of various factors [13]. We used mediation analysis to determine the mediating effect of spontaneous vaginal birth between midwife-led continuous labor and childbirth support and early initiation of breastfeeding [14].

## 3. Results

**Figure 1:**
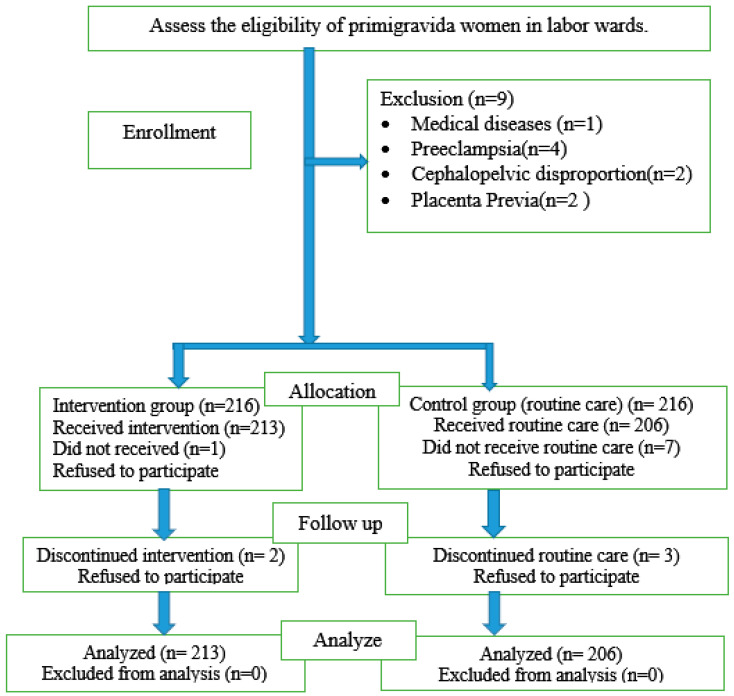
Consolidated Standards of Reporting Quasi-Experimental Flow Diagram.

The total sample size was 432, with 216 in the intervention group and 216 in the control group. However, three participants in the intervention group and ten in the control group did not participate for various reasons. Therefore, the final sample included 419 primigravida women—213 in the intervention group and 206 in the control group—in this study (Figure 1).

Eligibility of primigravida women (Figure 1).

The socio-demographic characteristics were similarly distributed between the intervention and control groups. Of the 419 primigravida women, most 132 in the intervention group and 148 in the control group—were aged 20–29 years (66.8%). Among the 293 (69.9%) urban residents, 139 were in the intervention group and 154 in the control group. One hundred ninety-eight primigravida women in the intervention group and 184 in the control group were Orthodox Christians (91.5%). Regarding marital status, 202 in the intervention group and 201 in the control group were married (96.5%). Twenty-seven primigravida women in the intervention group and seventy-six in the control group had attained college-level or higher education (24.8%). One hundred forty-one in the intervention group and ninety-four in the control group were housewives (56.1%). One hundred twenty primigravida women in the intervention group and 120 in the control group had a monthly family income less than or equal to 5000 birr (57.3%) (Table 1).

Midwife-led continuous labor support increased the rate of spontaneous vaginal delivery among primigravida women from 115 (55.8%) in the control group to 173 (81.2%) in the intervention group, resulting in a net increase of 25.4% (*p*-value < 0.001). Midwife-led continuous labor support resulted in 68.7% spontaneous vaginal deliveries, compared with 31.2% by cesarean section or instrumental delivery. It reduced the rate of episiotomy among primigravida women from 52 (25.2%) in the control group to 35 (16.4%) in the intervention group, with a net reduction of 8.8% (*p*-value < 0.026). Midwife-led continuous labor support increased early initiation of breastfeeding (within one hour) among primigravida women from 173 (56.8%) in the control group to 156 (73.3%) in the intervention group, with a net increase of 16.5% (*p*-value < 0.001) (Table 2).

### 3.1. Factors Associated with Early Initiation of Breastfeeding

Primiparous mothers living in urban areas were more than twice as likely to initiate early breastfeeding (within one hour) compared with those living in rural areas (AOR = 2.24, 95% CI: 1.20, 4.16). Primiparous mothers who were students or daily laborers were 64% less likely (AOR = 0.36, 95% CI: 0.17, 0.75) to initiate early breastfeeding (within one hour) compared with women whose occupation was being a housewife. Primiparous mothers who gave birth by spontaneous vaginal delivery were nearly 14 times more likely to initiate early breastfeeding (within one hour) compared with those who gave birth by cesarean section or instrumental delivery (AOR = 13.83, 95% CI: 8.21, 23.29) (Table 3).

### 3.2. Mediation Analysis

**Figure 2:**
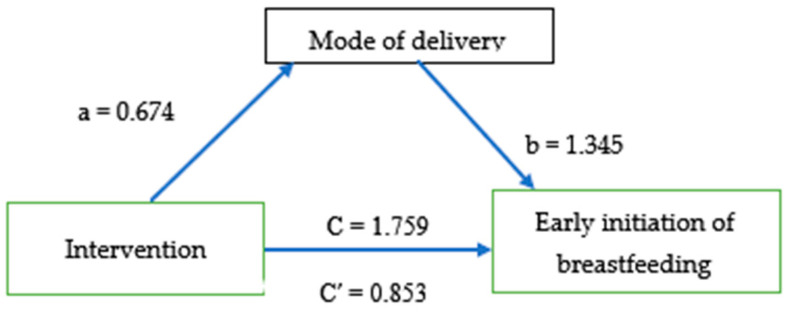
The mediating effect of mode of delivery on the relationship between midwife-led continuous labor and childbirth support and early initiation of breastfeeding.

Mode of delivery partially mediated the effect of midwife-led continuous labor and childbirth support (ß = 0.674, *p* < 0.05) and early initiation of breastfeeding. It also (ß = 1.345, *p* < 0.02) had a significant indirect effect on early initiation of breast feeding. The total effect of the mediation analysis was (ß = 1.759, *p* < 0.02) (Figure 2).

## 4. Discussion

Midwife-led continuous labor support increased the rate of spontaneous vaginal delivery among primigravida women from 55.8% in the control group to 81.2% in the intervention group, resulting in a net increase of 25.4%. The intervention reduced the rate of episiotomy from 25.2% in the control group to 16.4% in the intervention group, with a net reduction of 8.8%. The intervention increased early initiation of breastfeeding (within one hour) from 56.8% in the control group to 73.3% in the intervention group, with a net increase of 16.5%. Factors such as residence, education, occupation, and mode of delivery were associated with early initiation of breastfeeding among primiparous mothers.

Midwife-led continuous labor support is a component of the midwife-led model of care, which increases spontaneous vaginal birth and reduces the incidence of cesarean section [1,7,15]. This finding aligns with the present study, which found that midwife-led continuous labor support increased the rate of spontaneous vaginal delivery among primigravida women from 55.8% in the control group to 81.2% in the intervention group, resulting in a net increase of 25.4%. This is because midwife-led continuous care is guided by a philosophy that regards labor and childbirth as normal life events for women [16]. This is further supported by a clinical trial, which found that continuous labor and childbirth support for primigravida women increased the rate of spontaneous vaginal delivery [17]. This suggests midwife-led continuous labor and childbirth support reduces the rate of instrumental or cesarean section deliveries and may also reduce complications related to these births. It is time to focus on midwife-led, more humanistic, continuous supportive care during the labor and childbirth process [18]. Midwife-led continuous labor and childbirth support may improve childbirth outcomes [18,19]. Midwife-led support reduces cesarean section deliveries and interventions such as forceps use. These are distinct benefits of midwife-led care, which typically offers a more natural, less medicated, and psychosocially supportive approach to labor and childbirth, resulting in better intrapartum outcomes [2,20]. This supportive care could be implemented for all women in labor and childbirth in all maternity wards, as similar studies provide supporting evidence [21].

In the current study, the intervention reduced the rate of episiotomy from 25.2% in the control group to 16.4% in the intervention group, resulting in a net reduction of 8.8%. This finding is supported by evidence that continuous labor and childbirth support provided by midwives reduces the use of episiotomy during childbirth among primigravida women compared with controls [19]. It implies that implementing midwife-led continuous labor support in low-resource settings enhances positive intrapartum health outcomes [5,22]. This suggests that midwife-led continuous labor and childbirth support promotes hormone production, which facilitates strong uterine contractions and accelerates the natural childbirth process, thereby reducing the tradition of performing episiotomies [23]. It also helps prevent certain types of bleeding and sepsis-related complications, as well as reducing maternal discomfort in the postpartum period. By reducing episiotomy, promoting natural birth, and providing continuous support to women during labor and delivery, midwives can help ensure that childbirth remains a positive and empowering experience for all women [7,21]. This highlights the importance of promoting midwife-led support care and suggests future directions for policy and quality improvement practice. By reducing certain complications such as blood loss, pain and infections can be decreased [24].

Continuous midwife-led labor and childbirth support is associated with early initiation of breastfeeding [4]. In our study, the midwife-led continuous labor support intervention increased early initiation of breastfeeding (within one hour) from 56.8% in the control group to 73.3% in the intervention group, a net increase of 16.5%. Midwife-led continuous labor and childbirth support enhances the spontaneous childbirth process, prepares primiparous mothers psychosocially, and provides the opportunity to remain stable and free from complications. It enables primiparous mothers to initiate breastfeeding early. This finding is also supported by evidence that midwife-led care during labor and childbirth enhances physiological birth and early initiation of breastfeeding [25]. Early initiation of breastfeeding among primiparous mothers can be very challenging due to lack of experience and previous exposure. However, midwife-led continuous labor and childbirth support can address these challenges [26]. This implies that implementing midwife-led continuous labor support in low-resource settings enhances positive intrapartum health outcomes [22]. This shows that physical and psychosocial support enables mothers to begin breastfeeding early, which can prevent certain complications in the newborn and support growth and development.

The study suggested that primiparous mothers living in urban areas were more than twice as likely to initiate early breastfeeding (within one hour) compared with those living in rural areas. This is supported by evidence that mothers in urban areas are more likely to initiate early breastfeeding [27,28]. This may be explained by urban women being more likely to be educated and aware and to receive more information about the benefits of the early initiation of breastfeeding.

The current study showed that primiparous mothers who were students or daily laborers as their occupation were 64% less likely to initiate early breastfeeding (within one hour) compared with women whose occupation was being a housewife. This is supported by evidence that unemployed mothers are more likely to initiate breastfeeding early [29]. A possible explanation is that student or daily laborer primiparous mothers may not be psychologically stable, which could lead to fear and anxiety in the immediate postpartum period. These factors could negatively affect the initiation of early breastfeeding.

The study found that primiparous mothers who gave birth by spontaneous vaginal delivery were nearly 14 times more likely to initiate early breastfeeding (within one hour) than those who gave birth by cesarean section or instrumental delivery. Evidence suggests that vaginal delivery promotes the early initiation of breastfeeding [29]. This may be because midwife-led continuous support increases the rate of vaginal delivery and reduces complications such as obstetric bleeding, perineal laceration, and negative childbirth experiences associated with instrumental and cesarean section deliveries [6]. This suggests that primiparous mothers who gave birth vaginally were more likely to be physically and psychologically stable and to experience less immediate postpartum pain. This led to earlier initiation of breastfeeding, which could be further improved by implementing midwife-led continuous labor support care in all maternity wards. Spontaneous vaginal mode of birth partially mediated the effect of midwife-led continuous labor and childbirth support on the early initiation of breastfeeding. This implies that midwife-led continuous labor and childbirth support, both directly and indirectly through spontaneous per vaginal birth, enhanced the early initiation of breastfeeding [14]. Continuous labor support makes a paramount contribution and requires immediate action to implement it in all maternity wards.

## 5. Conclusions

Midwife-led continuous labor support increased the rate of spontaneous vaginal delivery and reduced the episiotomy rate. Midwife-led continuous labor support also significantly enhanced the early initiation of breastfeeding. Urban residency, education, and spontaneous vaginal delivery were associated with early initiation of breastfeeding among primiparous mothers. However, mothers who were students or daily laborers were less likely to initiate breastfeeding early. Spontaneous vaginal birth had a mediation effect.

These findings have clinical, policy, and program implications, supporting the scaling up of the intervention to other areas. Engaging these mothers in stable employment may encourage early initiation of breastfeeding. Midwife-led continuous labor and childbirth support should be provided at each stage of labor stages to enhance positive childbirth outcomes.

## Figures and Tables

**Table 1:** Socio-demographic characteristics of primigravida women in a midwife-led continuous labor support care study in northwest Ethiopia, 2024 (*n* = 419).

Variables	Categories	Status		Total	Percentage
		Intervention = 213	Control = 206		
Age	Less than 20	50	34	84	20
	20–29	132	148	280	66.8
	30 and above	31	24	55	13.2
Residence	Rural	74	52	126	30.1
	Urban	139	154	293	69.9
Religion	Muslim	15	20	35	8.4
	Orthodox	198	186	384	91.6
Marital status	Unmarried	11	5	16	3.8
	Married	202	201	403	96.2
Education level	Unable to read and write	54	20	74	17.7
	Read and write	45	12	57	13.6
	Primary school	49	37	86	20.5
	Secondary school	38	61	99	23.6
	College and above	27	76	103	24.6
Occupation	Housewife	141	94	235	56.1
	Merchant	26	30	56	13.4
	Government-employed	20	54	74	17.7
	Others *	26	28	54	12.8
Monthly family income	Less or equal to 5000 birr	120	120	240	57.3
	5001 to 10,000 birr	78	61	139	33.2
	Above 10,000 birr	15	25	40	9.5

Others * = student, daily labor, effect of midwife-led intervention.

**Table 2:** Effect of midwife-led continuous labor support on study outcomes between study groups among primigravida women in northwest Ethiopia, 2024 (*n* = 419).

Variables		Status		Total	*p*-Value
		Control = 206	Intervention = 213		
Mode of delivery	CS/Instrumental	91 (44.2)	40 (18.8)	131 (31.3)	<0.001
	SVD	115 (55.8)	173 (81.2)	288 (68.7)	
Episiotomy	No	154 (74.8)	178 (83.6)	332 (79.2)	<0.026
	Yes	52 (25.2)	35 (16.4)	87 (20.8)	
Breastfeeding initiation	Late	89 (43.2)	57 (26.8)	146 (34.8)	<0.001
	Early	117 (56.8)	156 (73.2)	273 (65.2)	

**Table 3:** Multivariable logistic regression analysis of factors associated with early breastfeeding initiation among primiparous mothers in northwest Ethiopia, 2024 (*n* = 419).

Variables		Breastfeeding Initiation		AOR% of CI	*p*-Value
		Late = 146	Early = 273		
Intervention status	Control	89	117	1.00	
	Intervention	57	156	0.89 (0.511–1.545)	0.675
Residence	Rural	50	76	1.00	
	Urban	96	197	2.24 (1.20–4.16)	0.011
Occupation	Housewife	76	159	1.00	
	Government-employed	19	37	0.96 (0.43–2.16)	0.935
	Merchant	23	51	1.21 (0.48–3.02)	0.675
	Others *	28	26	0.36 (0.17–0.75)	0.007
Delivery mode	CS/instrumental	94	37	1.00	
	SVD	52	236	13.83 (8.21, 23.29)	<0.001

Others * = students or daily laborers.

## Data Availability

This manuscript contains all data generated or analyzed in the course of the study. The data can be made available upon reasonable request from the corresponding author.

## Abbreviations

AOR = adjusted odds ratio; CI = confidence interval; CS = cesarean section; SVD = spontaneous vaginal delivery.
